# The HIV Landscape for Transgender Women Living in Southwest Asia and North Africa: A Scoping Review

**DOI:** 10.1002/jia2.70206

**Published:** 2026-09-18

**Authors:** Ala Koreitem, Sasha Abdallah Fahme, Parya Saberi

**Affiliations:** ^1^ Division of Prevention Science University of California San Francisco San Francisco California USA; ^2^ Center for Global Health Department of Medicine Weill Cornell Medicine New York New York USA; ^3^ Faculty of Health Sciences Epidemiology and Population Health Department American University of Beirut Beirut Lebanon

**Keywords:** HIV, mental health, Southwest Asia and North Africa, stigma, SWANA, transgender women

## Abstract

**Introduction:**

This scoping review aimed to characterize the prevalence and determinants of HIV acquisition among transgender women in Southwest Asia and North Africa (SWANA), to describe HIV preventive activities and measures, and to examine barriers to HIV and gender‐affirming care, including experiences of stigma, discrimination and mental health challenges among transgender women in the region.

**Methods:**

We searched PubMed, PsycINFO and CINAHL for any articles published through 12 June 2026, that included transgender women, used primary or secondary data and were published in Arabic or English. Studies meeting these criteria were screened in Covidence. Discrepancies were resolved following data extraction.

**Results:**

A total of 276 articles were identified (152 duplicates removed). After screening titles and abstracts, 49 full texts were assessed for eligibility. The articles included (*N* = 23) were primarily cross‐sectional studies (*N* = 20) conducted in Iran, Lebanon, Turkey, Morocco and Tunisia. Three were non‐randomized experimental studies; one intervention was designed specifically for transgender women, focusing on gender affirmation and social support. Cross‐sectional studies examined HIV prevention, sexual activities and mental health. Condomless receptive anal intercourse and low HIV testing rates were most common among those engaged in sex work, which was attributed to coercion, perceived low risk for HIV acquisition or fear of disclosing one's HIV status. Stigma, physical and sexual violence, anxiety, depression and suicidality were highly prevalent among transgender women in SWANA and served as barriers to accessing healthcare services, including HIV and gender‐affirming care.

**Discussion:**

This review highlights critical areas for trans‐specific interventions addressing transgender women's needs in SWANA, where HIV incidence remains on the rise.

**Conclusions:**

Future longitudinal research is needed to evaluate the effectiveness of interventions in reducing HIV rates, improving mental health outcomes and mitigating structural barriers hindering healthcare access among transgender women in SWANA.

## 1 Introduction

Transgender women are disproportionately impacted by HIV, with a 20‐fold greater risk of HIV acquisition compared to the general population worldwide [1]. Since 2010, global rates of new HIV acquisitions and HIV‐related mortality have declined by 40% and 51%, respectively, due to expanded access to HIV testing, pre‐exposure prophylaxis (PrEP) and antiretroviral therapy (ART) [2]. However, although some regions are experiencing a decrease in incidence, recent data indicate an escalating HIV epidemic in the Southwest Asia and North Africa (SWANA) region, where new acquisitions increased by 116% between 2010 and 2023 [3, 4].

Transgender women, men who have sex with men (MSM) and female sex workers are considered “key populations” and account for the majority of HIV cases in this region [3, 4]. A study conducted in 2016 by Kaplan et al. reported an HIV prevalence of 10% among transgender women in Lebanon, notably higher than the prevalence of 1.5%−3.6% among MSM in the country [5]. Research focusing on HIV among transgender women in SWANA remains scarce, hindering efforts to estimate the burden of HIV in this population accurately.

Globally, transgender women remain disproportionately underserved by HIV prevention and treatment efforts, and those living with HIV face additional challenges, making them less likely to be retained in care [6]. Key barriers to healthcare access include anticipated and experienced stigma and discrimination within healthcare settings, limited provider knowledge of transgender women's health needs, and a lack of culturally competent, gender‐affirming and trans‐specific health services [6, 7]. Furthermore, experiences of transphobia, stigma and discrimination can become internalized, increasing transgender women's risk for depression, post‐traumatic stress disorder (PTSD) and suicidality [8, 9, 10, 11]. Transgender women also face barriers to accessing gender‐affirming care, including gender‐affirming hormone therapy and surgery, which have been closely linked to improved quality of life and reduced mental health outcomes among transgender people [12, 13].

Lack of research contributes to limited interventions tailored to transgender women, underscoring the growing need for evidence‐based programmes that address HIV prevention and treatment disparities and the mental health burden faced by this population globally [14]. Community‐based methods incorporating peer navigation have shown promise in improving ART uptake, adherence and engagement in HIV care, as demonstrated in pilot studies conducted in North and Latin America [14, 15, 16]. More recently, interventions designed to enhance gender affirmation among transgender women have been developed and evaluated in countries such as Brazil and the United States [16, 17]. One example is Healthy Divas, a gender‐affirming intervention combining peer‐led individual sessions and group workshops, which showed improved engagement in HIV care among participants [16].

Although several interventions grounded in intersectional, healthcare empowerment and gender affirmation frameworks are currently being designed and piloted, many remain in early phases. They are still being assessed for efficacy and acceptability within their target populations. Even if proven effective, these models will require adaptation, translation and further evaluation across other communities of transgender women.

To date, there is limited research describing transgender women and HIV care efforts in the SWANA region. We conducted a scoping review to (1) identify the prevalence and determinants of HIV acquisition among transgender women in SWANA, (2) characterize HIV preventive measures among this population, (3) identify barriers that prevent transgender women in SWANA from receiving HIV care, including experiences of stigma, discrimination, and mental health challenges, and (4) highlight barriers to gender‐affirming care and their implications for sexual and mental health outcomes among transgender women in the region. Given the rapid rise in HIV incidence in the region, there is a critical need to better understand the needs of this key population to inform the development of trans‐specific HIV interventions that mitigate structural and cultural barriers and improve HIV prevention and treatment. This review identifies existing barriers to HIV care and gaps in the literature for transgender women in SWANA.

## 2 Methods

### 2.1 Search Strategy

This scoping review followed the Preferred Reporting Items for Systematic reviews and Meta‐Analyses extension for Scoping Reviews framework (PRISMA‐ScR) [18]. We searched the electronic databases PubMed, PsycINFO and CINAHL for peer‐reviewed literature published through 12 June 2026. Ethics committee approval was not required for this review article, as it only includes published articles. Consent was also not required, and a consent waiver was not obtained for the same reasons stated previously.

### 2.2 Eligibility Criteria

The studies that met the inclusion criteria were those that (1) included transgender women as part of the study population, (2) included primary data collection or analysed secondary data and (3) were conducted in one of the following countries/territories: Algeria, Bahrain, Comoros, Djibouti, Egypt, Iran, Iraq, Jordan, Kuwait, Lebanon, Libya, Morocco, Oman, Palestine (West Bank and Gaza), Qatar, Saudi Arabia, Somalia, South Sudan, Sudan, Syria, Tunisia, Turkey, United Arab Emirates or Yemen. The exclusion criteria were review articles and articles published in languages other than English or Arabic. We used the following key terms for our search: (transgender* OR transsexual*) AND HIV AND the countries/territories listed above.

### 2.3 Study Selection and Screening

Two authors (AK and SAF) conducted the literature search, with assistance from two librarians who helped develop a search strategy that met the format standards for each database. Two authors (AK and SAF) imported all references into Covidence, an online tool that facilitates the management of scoping reviews and systematic reviews [19]. Duplicate articles were removed either automatically by Covidence or manually as a first step. In accordance with the inclusion criteria, two authors (AK and SAF) independently screened titles and abstracts and conducted a full‐text review of the remaining articles. All discrepancies in the title/abstract screening and full‐text review stages were resolved through discussion and consensus among all three authors (AK, SAF and PS).

### 2.4 Data Extraction

Two authors (AK and SAF) used a standardized data extraction form in Covidence to independently extract data on aims, outcomes, study design, geographic location, intervention type and main findings from the final selected articles. Independent extraction was conducted to minimize bias in how the two authors interpreted and categorized the data, as discrepancies can arise when authors classify nuanced information differently. Discrepancies were resolved through discussion and consensus among all three authors (AK, SAF and PS). One author (AK) analysed the extracted data to identify recurring themes, grouped the final articles by study design, and summarized outcomes of interest and main findings. Data were presented in tables, with findings relevant to the themes identified in this review highlighted.

## 3 Results

### 3.1 Study Selection

**FIGURE 1:**
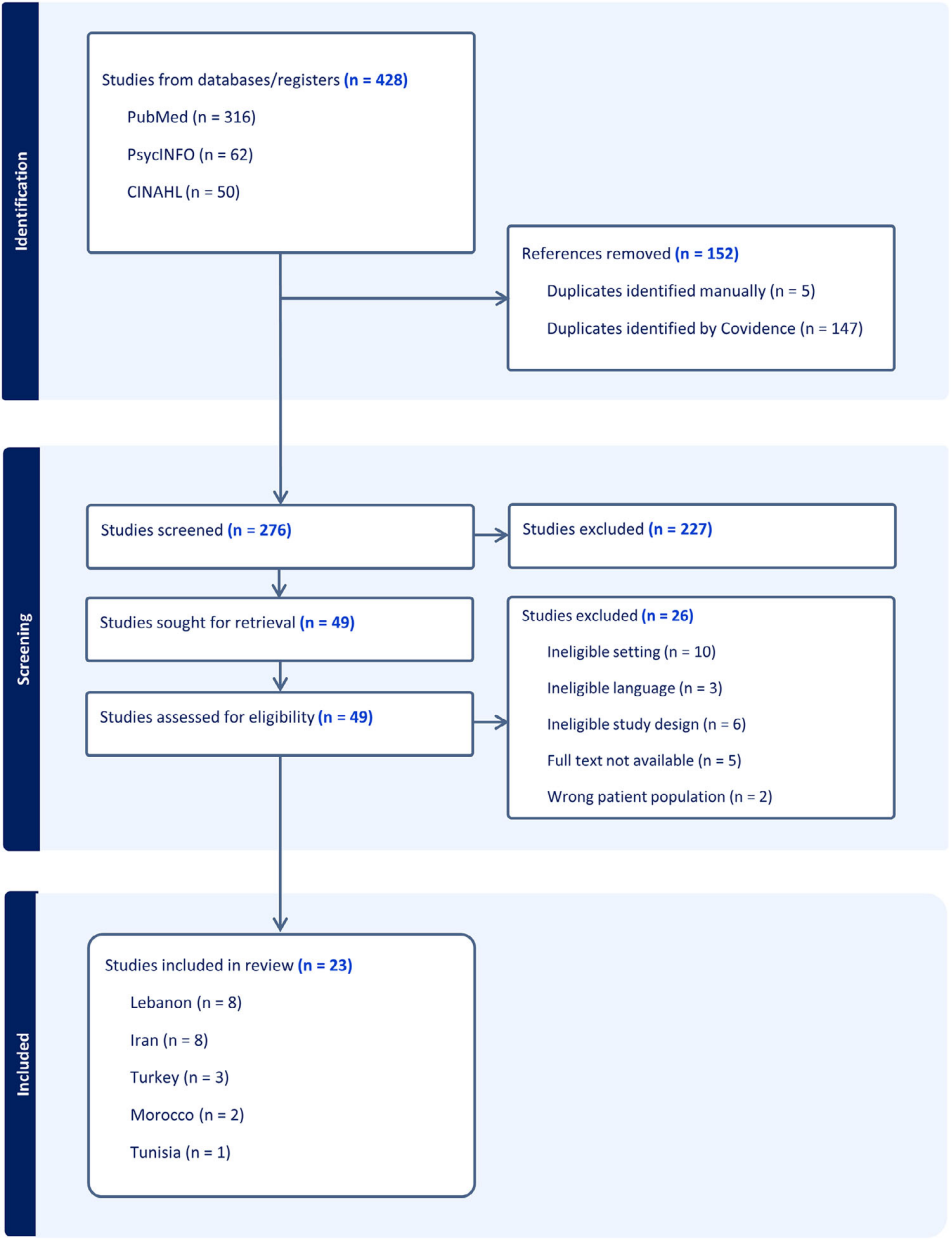
Preferred Reporting Items for Systematic Reviews and Meta‐Analyses extension for Scoping Reviews flowchart: flow diagram of study inclusion [18].

After removing 152 duplicates, 276 articles were retrieved from the three databases (Figure 1). Following title and abstract screening, 49 were retained for full‐text review. Reasons for exclusion are reported in Figure 1. A total of 23 studies met the eligibility criteria and were included in the review [20, 21, 22, 23, 24, 25, 26, 27, 28, 29, 30, 31, 32, 33, 34, 35, 36, 37, 38, 39, 40, 41, 42]. These studies are summarized in Tables 1 and 2. Table 1 summarizes the main characteristics of the included studies, grouped by study design, population and location. Table 2 presents the outcomes of interest and the main findings of the included studies.

**TABLE 1:** Characteristics of 23 research articles included in the scoping review.

Author (year)	Study design	Total number of participants	Study population	Location
Abou‐Rizk et al. (2016) [20]	Cross‐sectional study	116 (including two transgender adults)	People with HIV (PWH) in Lebanon who were registered with a non‐governmental organization (NGO) that provided access to medical care and social support services for PWH	Lebanon
Atuk et al. (2024) [21]	Qualitative research	55 (including 10 transgender women)	Healthcare providers, pharmaceutical industry employees, non‐governmental staff members, civil servants, HIV activists and transgender sex workers (TGSWs) working in Istanbul and Ankara	Turkey
Ben Moussa et al. (2024) [22]	Non‐randomized experimental study	3333 (including 62 transgender adults)	Female sex workers (FSWs), MSM and partners of newly diagnosed PWH in Morocco	Morocco
Bernier et al. (2019) [23]	Cross‐sectional study	300 (including 11 transgender adults)	PWH residing in Morocco who were in contact with a specific NGO	Morocco
Bozicevic et al. (2022) [24]	Secondary data analysis	N/A	Key populations (MSM, FSWs, intravenous drug users, transgender persons [TPs]), cases of HIV, cases of sexually transmitted infections (STIs) (diagnosed syndromically or etiologically)	22 countries in the Eastern Mediterranean region^a^
Clark et al. (2021) [25]	Cross‐sectional study	488 (including 45 transgender women)	Lebanese and Syrian MSM and transgender women in Beirut	Lebanon
Di Ciaccio et al. (2025) [26]	Qualitative research	19 (including two transgender women)	Sex workers (cisgender women and men, and transgender women) living in Tunisia	Tunisia
Eftekhar et al. (2020) [27]	Qualitative research	15	Transgender women from Tehran and Karaj	Iran
Gokengin et al. (2021) [28]	Mixed‐methods study involving programmatic mapping and key informant interviews	466 TGSWs in Istanbul and 96 TGSWs in Ankara	FSWs, TGSWs and MSM. “Key informants” were defined as those who directly or indirectly came into contact with the above key populations (e.g. taxi drivers, bar workers, club managers, police, healthcare providers and others)	Turkey
Hosseini Divkolaye et al. (2021) [29]	Cross‐sectional study	263 FSWs (including eight transgender sex workers)	FSWs in the southern parts of Tehran, Karaj and suburban regions	Iran
Ibrahim et al. (2016) [30]	Cross‐sectional study	40 (20 transgender women and 20 controls)	Transgender Lebanese women and age‐ and sex‐matched controls	Lebanon
Kaplan et al. (2020) [31]	Non‐randomized experimental study	16	Transgender women in Beirut	Lebanon
Kaplan et al. (2019) [32]	Mixed‐methods pilot intervention study	16	Transgender women in Beirut	Lebanon
Kaplan et al. (2016) [33]	Cross‐sectional study	53	Transgender women in the greater Beirut area	Lebanon
Kaplan et al. (2015) [34]	Qualitative research	10	Transgender women in Lebanon	Lebanon
Kasapoǧlu and Kus (2008) [35]	Qualitative research	16 (including two transgender adults)	PWH	Turkey
Khodakhah and Jamshidimanesh (2023) [36]	Cross‐sectional study	96 (including 23 transgender women)	TPs of both genders in Tehran	Iran
Moayedi‐Nia et al. (2019) [37]	Cross‐sectional study	104	Transgender women in Tehran	Iran
Moradi et al. (2022) [38]	Mixed‐methods cross‐sectional study	930 participants (including 49 transgender adults)	Iranian key populations, including MSM, FSWs and TPs visiting HIV self‐test centres	Iran
Nadoushan et al. (2021) [39]	Cross‐sectional study	58	Adults with “gender identity disorder” who were referred to the Tehran Institute of Psychiatry for treatment	Iran
Nematollahi et al. (2022) [40]	Cross‐sectional study	127	Transgender women in Tehran and Shiraz	Iran
Orr et al. (2022) [41]	Cross‐sectional study	549 (including 16 transgender women)	Lebanese and displaced Syrian MSM and transgender women in Beirut	Lebanon
Saadat et al. (2024) [42]	Qualitative research	22	Transgender women sex workers in Iran	Iran

Abbreviations: FSWs, female sex workers; MSM, men who have sex with men; NGO, non‐governmental organization; PWH, people with HIV; STIs, sexually transmitted infections; TGSWs, transgender sex workers; TPs, transgender persons.

^a^ Afghanistan, Bahrain, Djibouti, Egypt, Iran, Iraq, Jordan, Kuwait, Lebanon, Libya, Morocco, Oman, Pakistan, Palestine, Qatar, Saudi Arabia, Somalia, Sudan, Syria, Turkey, United Arab Emirates, Yemen.

**TABLE 2:** Outcomes of interest and relevant findings among 23 research articles included in the scoping review.

Author (year)	Outcomes	Relevant findings
Cross‐sectional studies (*n* = 20)		
Quantitative studies (*n* = 12)		
Abou‐Rizk et al. (2016) [20]	Complementary and alternative medicine (CAM) use among PWH	Other findings: One transgender (TG) participant used CAM, and one did not. Although TG participants were included, the analyses were not stratified by sub‐populations.
Bernier et al. (2019) [23]	Self‐esteem and factors associated with living with HIV	Mental health: The mean self‐esteem score was 2.0 for TG participants, which was lower than that of cisgender women and men.
Bozicevic et al. (2022) [24]	HIV prevalence	The only country with surveillance data on TG populations is Pakistan (outside the scope of review). The authors attributed the severe lack of HIV data on TG people in other countries to a “lack of social and legal recognition of gender‐diverse persons and social marginalization.”
Clark et al. (2021) [25]	Stigma, displacement stressors and psychiatric morbidity	Mental health: Transgender women (TW) were significantly more likely to have depression, anxiety and PTSD than cisgender persons. Syrian TW experienced higher levels of depression and PTSD than Lebanese‐born TW. Stigma/violence: Syrian TW faced stigma‐ and displacement‐related stressors as a sexual or gender minority and a displaced person, with sexual minority‐related discrimination and assault, and internalized sexual minority stigma associated with greater depression.
Hosseini Divkolaye et al. (2021) [29]	Factors associated with sexual and physical violence against FSWs	Though the study included TW, none of the analyses explicitly included this sub‐population.
Ibrahim et al. (2016) [30]	Psychiatric comorbidities and mental health needs of TG persons	Mental health: Active suicidal thoughts (55%), major depressive episodes (45%), generalized anxiety disorder (5%) and PTSD (5%) were reported among TG participants. Suicidal ideation and Axis I disorders were significantly more common among TG participants than controls.
Kaplan et al. (2016) [33]	HIV prevalence and demographic determinants of CRAI	HIV prevalence: 10% (*n* = 4). Many participants had never been tested for HIV (40%), and few had been tested in the past year (17%). HIV prevention: Sex work, being fully out at work or school and being arrested due to their TG identity were associated with greater odds of CRAI. History of sexual abuse was protective against CRAI. Stigma/violence: Many experienced discrimination (98%) and physical abuse (68%), and lacked familial support (68%). About half were sexually abused (49%). Mental health: Current depression (62%), prior suicide attempts (42%), suicidal ideation (21%) and being uncomfortable with one's gender (42%) were common among participants. Gender affirmation: Gender affirmation practices included hormone use (52.8%) and surgery (18.9%).
Khodakhah and Jamshidimanesh (2023) [36]	Sexual/reproductive health‐related measures in TG individuals	HIV prevention: 77% of TG participants had never tested for HIV; 73% had never used condoms; 76% perceived no threat of CRAI. Stigma/violence: 37% of TG participants reported experiencing stigma and discrimination; 38% experienced sexual violence; 75% experienced violence; 86% reported not feeling good about having to be referred to a health centre. Mental health: 25% reported suicidal ideation; 73% reported attempting suicide. Gender affirmation: All 96 TG participants completed hormone therapy; of those, 45% underwent GAS.
Moayedi‐Nia et al. (2019) [37]	HIV prevalence and sexual activities among TW	HIV prevalence: 1.9% (*n* = 2). HIV prevention: Engaging in CRAI was highly prevalent among TW participants, despite low rates of HIV testing, and given that most never or rarely use a condom. Prevalence of condom use was highest for those having sex with a paying partner (53%). Stigma/violence: 25% reported experiencing sexual abuse. Gender affirmation: 33% of participants completed GAS.
Nadoushan et al. (2021) [39]	Sexual activities among TG individuals associated with HIV exposure	HIV prevalence: 0% (93% consented to test for HIV). HIV prevention: Among those who do not have sex with women, almost half (45%) said they do not use a condom. No one reported a history of using IV drugs, or sex after using drugs, or sex to obtain drugs.
Nematollahi et al. (2022) [40]	Sexual activities of TW and their vulnerability to STIs	HIV prevalence: 1.6% (*n* = 2). HIV prevention: Most participants had never had an STI test (87%) or an HIV test (72%). Reasons for not having an HIV test were not considering themselves at risk. Only 30% of participants recognized all mechanisms of HIV transmission, yet most felt they were not at risk of HIV acquisition (88%). Few reported that they “always” used a condom (18%), and 34% reported never using one. Reasons for not using a condom were not having one, decreased pleasure and sexual partner rejection. Gender affirmation: 26% underwent vaginoplasty.
Orr et al. (2022) [41]	Behaviours associated with HIV exposure	The study did not stratify results by population (separating TW from cisgender MSM).
Mixed‐methods study (*n* = 2)		
Gokengin et al. (2021) [28]	Size and distribution of key populations	HIV prevention: 15,780 TGSWs were estimated in Istanbul and 1770 in Ankara. The mean sexual network size for TGSWs was higher than that of cisgender FSWs but lower than that of MSM. Most TGSWs operated at street spots rather than bars, nightclubs and cafes. Stigma/violence: TW in Turkey faced barriers to other employment, violence, harassment and discrimination, and would resort to sex work as a profession instead.
Moradi et al. (2022) [38]	Feasibility and acceptability of using HIV self‐tests	HIV prevalence (all study participants): 1.5% (not stratified by group, so it is unclear how many TW had a new HIV diagnosis). HIV prevention: Most participants had no knowledge of HIV self‐tests (73%) and had never taken one (96%). Acceptability of self‐tests was high (98%). Most said they would use them again or recommend them.
Qualitative studies (*n* = 6)		
Atuk et al. (2024) [21]	Medical violence and transphobia experienced by TGSWs accessing medical care	HIV prevention: TW in Turkey found themselves obligated to enter sex work as other professional areas were not accessible to them. Stigma/violence: TW were denied care by medical professionals based on both gender identity and perceived risk of infectious disease transmission due to assumptions regarding sex work. While TW experienced both gender identity and sex work stigma within healthcare settings, irrespective of their HIV status, those who were HIV positive were frequently denied care altogether, including gender‐affirming care, due to fears of nosocomial transmission.
Di Ciaccio et al. (2025) [26]	Socioeconomic constraints on sex workers’ agency during the COVID‐19 pandemic	HIV prevalence: One of two TW participants was living with HIV (5.3% of the total sample). HIV prevention: A TW sex worker reported being forced to engage in CRAI with their clients despite fearing STI acquisition. Economic precarity prevented participants with HIV from accessing HIV treatment due to fears of COVID‐19 infection and transportation costs. Stigma/violence: HIV status acted as a barrier to COVID‐19 vaccination; seeking vaccine information risked inadvertent HIV status disclosure and experiencing stigma from vaccination staff. Mental health: TW reported mental health challenges around living with family during the pandemic, fearing inadvertent disclosure of their HIV status, and gender and/or sexual identities.
Eftekhar et al. (2020) [27]	Factors associated with sexual activities linked to HIV exposure in TW	HIV prevention: Common sexual practices among TW included CRAI with multiple unfamiliar partners, at a partner's request or to receive money, protection and/or shelter. Many did not perceive themselves at a high risk of HIV acquisition. Some were fatalistic about HIV, believing that it did not matter because they believed they already did not have a future. Gender affirmation: Several participants engaged in sex work to fund cosmetic surgeries that enhanced their feminine appearance.
Kaplan et al. (2015) [34]	HIV/AIDS‐related risk and resilience among TW	HIV prevention: Coercive sex was common among participants engaging in sex work. Some described engaging in CRAI if a client paid more not to use a condom. For many who were not living at home, financial safety was a motivation for engaging in sex work. Stigma/violence: Participants experienced stigma, discrimination and transphobia. Most reported experiencing physical or sexual violence. Mental health: Most participants noted having a current or prior “mental health problem.” Half had considered or attempted suicide. Gender affirmation: Participants living at home experienced tension and frustration of a double life because they were restricted from expressing their gender identity.
Kasapoǧlu and Kus (2008) [35]	Gender stigma	HIV prevalence: Two participants were TW with HIV. Stigma/violence: Authors found that TGSWs with HIV are particularly vulnerable in Turkey and may be exposed to violence upon disclosure of their diagnosis. A participant expressed fear over disclosing her HIV status in her social life and workplace due to perceived stigmatization and possible danger. Another lost friendships with other TW after being diagnosed with HIV because they were fearful of contracting it through “sitting side‐by‐side” or drinking water from the same glass.
Saadat et al. (2024) [42]	TW sex workers’ access to sexual healthcare	HIV prevention: Financial cost and lack of insurance coverage led participants to engage in sex work. The lack of post‐GAS care and knowledgeable providers of TG health was a major obstacle to accessing care. Participants engaging in sex work were less likely to use condoms due to social pressure or coercion. Stigma/violence: Reports of stigma, discrimination and poor access to SH services were common among TW sex workers. Some avoided seeking services, fearing doctors would report them to the police. Reports of physical, sexual and psychological violence were also common. Mental health: Some reported drug use as a coping mechanism for dealing with depression and other mental health challenges.
**Non‐randomized experimental studies (*n*** = **3)**		
Quantitative studies (*n* = 2)		
Ben Moussa et al. (2024) [22]	Acceptability and feasibility of HIV self‐testing distribution models	Duration of study: 6 months The study did not stratify results by population. HIV prevalence: The rate of HIV positivity was 1.2% among MSM and FSWs and 4.3% among partners of PWH. HIV prevention: All HIV self‐testing distribution models were feasible and effective in reaching people who had never been tested. 86.2% of participants in the arm into which most TG participants were allocated (2.4%; *n* = 59) said they were willing to buy and use an HIV self‐test.
Kaplan et al. (2020) [31]	Feasibility, acceptability and impact of a pilot intervention on mental and sexual health outcomes	Duration of study: 2 months HIV prevalence: 6% (*n* = 1). Mental health: There was a significant association between war event exposure and higher anxiety. At 3 months post‐intervention, higher social cohesion was significantly correlated with less suicidal ideation; more community connectedness was significantly associated with lower depression. Compared with baseline, seven of 13 participants had improved
		gender affirmation satisfaction scores; nine of 13 had improved community connectedness and social cohesion scores. HIV prevention: There was an increase in the proportion of participants testing for HIV between the 6 months prior to the intervention (47%) and following it (73%). Lack of access/awareness/support may have acted as a barrier to testing prior to the intervention.
Mixed‐methods studies (*n* = 1)		
Kaplan et al. (2019) [32]	Feasibility and acceptability of a behavioural group support intervention	Duration of study: 2 months HIV prevalence: 6% (*n* = 1). HIV prevention: 31% of participants reported current sex work, and 6% reported prior sex work. Other findings: The retention rate for participation in the 6‐week “Baynetna” intervention was high at 94% (15 out of 16 participants) and 81% at the 3‐month post‐test follow‐up assessment. This suggests excellent feasibility and acceptability of the group intervention.

Abbreviations: CRAI, condomless receptive anal intercourse; GAS, gender‐affirming surgery; MSM, men who have sex with men; PTSD, post‐traumatic stress disorder; SH, sexual health; TG, transgender (transgender women and transgender men); TW, transgender women.

### 3.2 Study Characteristics

Most articles were cross‐sectional (*n* = 20; 87%) [20, 21, 23, 24, 25, 26, 27, 28, 29, 30, 33, 34, 35, 36, 37, 38, 39, 40, 41, 42]. Of those, 12 were quantitative studies (60%) [20, 23, 24, 25, 29, 30, 33, 36, 37, 39, 40, 41], six were qualitative studies (30%) [21, 26, 27, 34, 35, 42] and two were mixed‐methods studies (10%) [28, 38]. Three articles were non‐randomized experimental studies (13%) [22, 31, 32]; of those, two were quantitative studies (67%) [22, 31], and one was a mixed‐method pilot intervention study (33%) [32]. Sample sizes ranged from 10 to 3333 participants. Nearly all studies recruited participants from clinics/hospitals or local nonprofit organizations. Studies were conducted primarily in Lebanon (*n* = 8; 35%) [20, 25, 30, 31, 32, 33, 34, 41] and Iran (*n* = 8; 35%) [27, 29, 36, 37, 38, 39, 40, 42]. Three studies were conducted in Turkey (13%) [21, 28, 35], two in Morocco (9%) [22, 23] and one in Tunisia (4%) [26]. One study (4%) examined 22 countries in the World Health Organization (WHO) Eastern Mediterranean region [24]. Only 11 out of 23 articles (48%) focused on transgender women exclusively [27, 28, 30, 31, 32, 33, 34, 37, 39, 40, 42]. Recurring themes observed in this scoping review included activities associated with HIV exposure and HIV prevention, experiences of stigma and violence, and mental health.

### 3.3 HIV Prevalence and Testing

Among the included studies, six (26%) reported on HIV prevalence among transgender women in Iran, Lebanon and Tunisia, which ranged from 1.6% to 10% of study samples [26, 31, 37, 38, 40, 43]. Among studies reporting HIV prevalence, half relied on participant self‐report (*n* = 3, 50%) [26, 31, 40]; two used HIV testing (*n* = 2, 33%) [33, 38], and one estimated prevalence through dried blood spot sample analysis (*n* = 1, 17%) [37]. One qualitative study (*N* = 16) conducted in Turkey reported that all transgender participants (*n* = 2; 13% of the study sample) were living with HIV [35]. Of the six studies reporting prevalence, four (67%) assessed HIV testing among study participants [31, 38, 40, 43]. These studies consistently revealed that awareness of and/or access to HIV testing was notably low among transgender participants. For example, Kaplan et al. (*N* = 16) found that just 47% (*n* = 7) of transgender women in their sample had ever been tested for HIV prior to their intervention [31]. Nematollahi et al. (*N* = 127) found that, although 88% (*n* = 112) of participants did not consider themselves at risk of acquiring HIV, most participants (*n* = 92, 72%) had never taken an HIV test in the past, and only about 18% reported consistent condom use [40]. Participants cited fear of abuse and perceived low personal risk of HIV acquisition as key reasons for not engaging in preventive measures such as HIV testing. Similar to Nematollahi et al., Khodakah and Jamshidimanesh (*N* = 96) reported that 77% (*n* = 74) of their sample of transgender participants had never tested for HIV. However, the authors did not assess HIV prevalence among their sample [36].

Only two studies (10%) evaluated the feasibility and acceptability of HIV self‐testing in Iran and Morocco [22, 38]. In Iran, Moradi et al. (*N* = 44) found that 73% (*n* = 32) of transgender persons in their sample were unaware of HIV self‐tests, and 96% (*n* = 42) said they had never used one before. However, nearly all participants (*n* = 40, 98%) expressed that HIV self‐testing would be an acceptable and feasible prevention method. Similarly, an intervention study assessing the usability and feasibility of their HIV self‐testing kits over 6 months (*N* = 62) also found high self‐test acceptability rates (80%–90%) among study participants from key populations in Morocco.

### 3.4 HIV Prevention

Nine studies (39%) conducted in Iran, Lebanon and Turkey evaluated sexual activities among transgender women that may increase HIV acquisition, with condomless receptive anal intercourse (CRAI) being the most reported sexual activity [21, 27, 33, 34, 36, 37, 39, 40, 42]. In Iran, Nematollahi et al. (*N* = 127) found that only 5.5% (*n* = 7) of participants reported consistent condom use during anal sex, and just 18% (*n* = 20) stated they always used condoms [40]. Another study (*N* = 96) found that most participants had never used condoms (*n* = 70, 73%) and perceived no threat to CRAI (*n* = 73, 76%) [36]. Similarly, a study (*N* = 104) also conducted in Iran reported that approximately 56% (*n* = 41) of participants never or rarely used condoms during sex [37]. Among those engaging in sex work (*n* = 11, 15%), condom use during receptive anal intercourse was reported by only about half of them.

Engaging in sex work emerged as another commonly reported characteristic among this population, which was associated with increased rates of CRAI [26, 33, 42]. For instance, in a qualitative study conducted in Lebanon (*N* = 10), one participant expressed that she would engage in condomless intercourse if a client offered more money [34]. This was also reported in Saadat et al.’s qualitative study in Iran (*N* = 22), in which a participant expressed difficulty negotiating condom usage due to social pressure or coercion [42]. Several studies (*n* = 4, 17%) identified a lack of familial and/or financial support as key structural drivers of sexual activities associated with HIV exposure [26, 33, 34, 42]. In fact, financial safety was found to be a significant motivation for engaging in sex work, especially among transgender women who were no longer living with their parents [34].

None of the articles included in this review addressed PrEP or ART uptake and/or access among transgender women in SWANA.

### 3.5 Stigma, Violence and Mental Health Challenges

Nine studies (39%) described experiences of stigma and discrimination among transgender women in Iran, Lebanon, Turkey and Tunisia in relation to their gender identity [21, 25, 26, 28, 33, 34, 35, 36, 42]. Fear of disclosing one's HIV status due to anticipated stigma and potential violence was a recurring theme in three of these studies (33%) [26, 35, 40]. Experiences of physical and sexual abuse were explored and reported in seven studies (78%) conducted in Iran, Lebanon and Turkey [28, 33, 34, 35, 36, 37, 42]. For example, a qualitative study in Lebanon found that 9 out of its 10 participants (90%) had previously experienced physical or sexual violence [34]. Another study in Lebanon (*N* = 53) reported that about 70% (*n* = 36) of participants experienced physical abuse, and about 50% (*n* = 26) were sexually abused, specifically due to their transgender identity [33]. Similarly, a cross‐sectional study in Iran (*N* = 96) reported that 75% (*n* = 72) of their transgender participants experienced violence and 38% (*n* = 36) experienced sexual violence [36]. Transgender women who engage in sex work are particularly vulnerable to violence, especially upon disclosure of their HIV status [35].

The role of intersecting forms of stigma was also explored in two studies (9%), which found that multiple marginalized identities (such as one's sexual and gender identities and country of origin) can compound to exacerbate experiences of discrimination [25, 26]. Clark et al. (*N* = 488) reported that displaced Syrian transgender women in their study sample faced unique and intersectional stressors related to their status as a displaced person and as a gender minority compared to Lebanese‐born transgender women [25]. These participants also experienced significantly higher levels of depression and PTSD compared to Lebanese‐born participants [25].

Eight studies (35%) assessed mental health outcomes among transgender women in Lebanon, Iran and Tunisia [25, 26, 30, 31, 33, 34, 36, 42]. One study reported that experiences of sexual minority‐related discrimination and assault, along with internalized sexual minority and HIV‐related stigma, were significantly associated with higher levels of depression among transgender women participants in Lebanon [25]. A study (*N* = 40) in Lebanon found that 45% (*n* = 9) of their transgender participants (*n* = 20) had experienced a major depressive episode [30]. Similarly, another study (*N* = 53) in Lebanon found that about 62% (*n* = 33) of participants reported feeling depressed at the time of the study, and 42% (*n* = 22) reported a prior suicide attempt [33].

One study (*N* = 22) in Iran noted that three participants reported drug use as a coping mechanism for dealing with depression and other mental health challenges [42]. Social support from other transgender women was identified as a protective factor in improving mental health outcomes and promoting overall wellbeing [31]. In Kaplan et al.’s pilot intervention study, the authors implemented “Baynetna,” an intervention consisting of 6 weekly trans‐facilitated group sessions focused on gender affirmation and peer‐based social support, which showed a high retention rate of 94% among transgender women in Beirut [31, 32]. The authors found that increased community connectedness and social cohesion among transgender women in Lebanon were significantly associated with lower depression scores and fewer suicidal thoughts, respectively [31].

### 3.6 Gender Affirmation and Healthcare Access

Five studies (24%) examined gender dysphoria and the prevalence of gender‐affirming surgeries (GAS) among transgender women in Iran and Lebanon [27, 33, 37, 40, 42]. In one study (*N* = 53) conducted in Lebanon, about 42% (*n* = 22) of the sample reported being uncomfortable with their gender identity, whereas about 53% (*n* = 25) had used hormones for gender confirmation, and only about 19% had undergone GAS [33]. The rate of completed GAS varied by study and appeared to differ by the location of the study [27, 33, 37, 40]. Studies conducted in Iran reported higher percentages of completed surgeries than those in Lebanon (33% vs. 19%) [33, 37]. This difference may be attributed to the Iranian government's legal and financial support for gender‐affirming procedures [37].

Nonetheless, barriers to healthcare services persist in Iran. A qualitative study (*N* = 22) reported that discrimination, a lack of providers’ knowledge and the high cost of health services prevented transgender women sex workers from seeking care for their HIV and sexual health needs [42]. In Tunisia, transgender women living with HIV who engage in sex work described economic precarity as an additional barrier to accessing HIV treatment during the COVID‐19 pandemic [26]. They also described fears of experiencing stigma by COVID‐19 vaccination staff upon disclosure of their HIV status, preventing them from getting vaccinated [26].

Familial support was assessed in four studies (17%) conducted in Iran, Lebanon and Tunisia [26, 33, 34, 42]. A qualitative study conducted in Tunisia (*N* = 19) found that transgender women participants who engaged in sex work (*n* = 2, 11%) reported mental health challenges relating to living with family during the COVID‐19 pandemic and generally feeling socially isolated [26]. These participants described fearing the inadvertent disclosure of their HIV status, gender and/or sexual identities by family members but felt forced to live at home due to increased economic precarity, exacerbated by a decline in clients seeking sex workers during the pandemic [26]. In Lebanon, Kaplan et al. (*N* = 53) found that 68% of participants lacked support from their families as related to their gender affirmation [33]. Similarly, participants in Saadat et al.’s study (*N* = 22) in Iran described experiencing social isolation from family and friends due to relying on sex work to meet daily needs [42]. In another study (*N* = 10) in Lebanon, participants living at home expressed frustration over being unable to express their gender identity freely [34].

## 4 Discussion

This scoping review aimed to examine existing literature on the prevalence and determinants of HIV acquisition, HIV preventive measures, and barriers to HIV and gender‐affirming care among transgender women in the SWANA region, including the roles of stigma and mental health challenges. To our knowledge, this is the first scoping review to synthesize evidence on the experiences and characteristics of transgender women in SWANA. The 23 studies we identified for this review reveal critical areas to target to address the health disparities faced by transgender women in the region.

Our search brings to light a significant gap in research estimating the prevalence of HIV among transgender women in SWANA. Aside from studies conducted in Lebanon, Iran and Tunisia, there are no other studies that directly estimate HIV prevalence among this population. Current efforts in the region focus largely on groups like MSM and female sex workers but fail to centre their research on transgender women populations as a distinct group [28]. This is alarming considering transgender women in other regions of the world, such as Latin America, have been shown to be among the most vulnerable to HIV [1, 44, 45]. HIV surveillance efforts can be disrupted as a result of protracted conflict, sociopolitical and economic crises, and forced population displacement across the region, affecting key populations disproportionately [46]. Access to healthcare services like HIV prevention and treatment is also hindered in humanitarian crises [46, 47]. Displaced transgender women and MSM have been shown to resort to CRAI, sex work, and substance use as coping mechanisms and to have an increased prevalence of displacement‐related stressors and psychiatric comorbidities [25, 31, 41, 46]. These gaps in services increase the susceptibility of transgender women to HIV acquisition and directly contribute to the rise in HIV incidence in the region.

Several studies in our review documented the high prevalence of CRAI among this population, often in the context of sex work and frequently driven by fear of violence and repercussions, and a perceived low risk of HIV acquisition. A disconnect between engaging in activities that increase exposure to HIV and a lack of concern or awareness of being at risk of acquiring HIV was evident across multiple studies [27, 36, 40]. This pattern is not unique to SWANA; high prevalence of CRAI and suboptimal uptake of HIV testing has been observed among transgender populations in other regions, such as the United States, behaviours that are also due to low‐risk perception of HIV acquisition and fear of HIV‐related stigma [48, 49, 50]. Thus, future programming should focus on increasing awareness of the risks associated with condomless sex, particularly among transgender women who engage in sex work.

With respect to sex work, its criminalization and resulting stigma and violence force many to operate through hidden networks without legal protection, creating precarious working conditions that make negotiating condom use more difficult and potentially unsafe [26, 28, 51, 52]. Evidence suggests that the decriminalization of sex work, coupled with the implementation of effective and fair judiciaries, is essential to reducing police violence, expanding access to condoms, improving condom negotiation, and ultimately reducing HIV prevalence among this population [51, 52].

Another critical area to consider is the heightened vulnerability of transgender women in SWANA to stigma and violence related to their sexual orientation and gender identity, including in healthcare settings. Our review sheds light on these experiences, which are often exacerbated upon disclosure of one's HIV status, especially for those engaging in sex work and those interacting with healthcare providers. The lack of qualified providers with experience in transgender health further contributes to transgender women's experiences of healthcare stigma and medical violence across SWANA countries [21, 42, 53]. The intersecting identities of transgender women make them especially vulnerable to discrimination and violence compared to other groups [54]. Results from Clark et al.’s study further highlight the need to address intersectional stigma, where overlapping systems of oppression, such as transphobia, racism and classism, compound and negatively affect health outcomes [25, 26, 54]. Findings from the studies in this review align with existing literature examining experiences of intersectional stigma and its role in exacerbating health inequities among transgender women globally [11, 15, 55, 56].

Our review also demonstrates the substantial burden of mental health outcomes faced by transgender women in SWANA, such as depression, anxiety, PTSD and suicidality. This finding is consistent with research from the United States and other parts of the world, showing poor mental health outcomes in this population [11, 14, 57]. This evidence points to an urgent need for additional research on interventions that directly address the mental health needs of transgender women in SWANA.

Our literature search highlights that there is a severe lack of research focused on the specific needs, experiences and health outcomes of transgender women in SWANA. Most notably, data from longitudinal studies and intervention trials are particularly scarce, with most of the studies in this review being cross‐sectional or formative. In addition, those that were longitudinal were conducted within short timeframes, ranging from 2 to 6 months [22, 31, 32].

In other regions of the world, peer navigation‐based trials have successfully demonstrated improved HIV care engagement among transgender women [14, 16]. However, our search revealed very few culturally adapted programmes tested among transgender women in SWANA. Kaplan et al.’s pilot is one of the few examples of a successful intervention being designed and implemented specifically for transgender women living in the region [31]. The “Baynetna” intervention was adapted to the Lebanese context from an existing intervention (“TransAction”) originally developed in Los Angeles, California. “Baynetna” was designed with a focus on gender affirmation and peer‐based social support within transgender communities in Beirut. The intervention showed a high retention rate of 94%, improved HIV testing uptake, community connectedness, social cohesion and gender affirmation satisfaction scores among participants [31]. The intervention's success among this group suggests it may demonstrate similar feasibility and acceptability among other communities of transgender women in SWANA.

Only half of the articles selected for this scoping review had transgender women as their primary focus. In most cases, transgender women were grouped with MSM or female sex workers and comprised only a small portion of the total sample. Several studies failed to stratify findings by sub‐population or excluded transgender women from subgroup analyses altogether. Grouping transgender women with other communities is a recurring issue among studies conducted both within and outside of SWANA that contributes to the erasure of trans‐specific issues, such as gender affirmation [14].

In addition, the studies meeting eligibility for this review were only available from five countries within SWANA (Iran, Lebanon, Turkey, Morocco and Tunisia). Yet, previous evidence has revealed a concentrated HIV epidemic among female sex workers in Djibouti, Somalia and South Sudan [58]. The disparities in evidence among several countries in SWANA raise serious concerns about the status of the epidemic in SWANA, as this review could not capture the true burden of disease among key populations in most countries in the region.

Bozicevic et al. have attributed the scarcity of HIV data on transgender people in the WHO Eastern Mediterranean region to both social marginalization and a lack of social and/or legal recognition of gender‐diverse individuals [24]. Iran represents a unique case, where transgender women are legally recognized as a community and have access to government‐funded hormonal and gender‐affirming medical procedures [27]. This is a result of gender dysphoria being framed as a medical condition requiring treatment [37]. The legalization and funding of GAS by the Iranian government aimed to push more transgender individuals to undergo the procedure, obtain official documents with their updated gender and eliminate gender dysphoria, rather than to affirm their gender identity [37]. Yet, despite having legal rights, transgender women in Iran continue to face significant social stigma, discrimination and mental health challenges that cannot be addressed without appropriate and culturally sensitive interventions specifically tailored to this population [27].

Similar to Iran, Lebanon and Turkey allow gender and name changes under strict conditions, such as undergoing GAS [59]. Nevertheless, punitive laws and the criminalization of sex work, same‐sex relationships and transgender identities remain prominent across numerous countries in the region, including some that enforce the death penalty [51, 58]. These laws, when coupled with existing conservative cultural and religious norms, further impede HIV prevention efforts [51, 58]. They deter transgender women from seeking HIV prevention and treatment services out of fear of legal repercussions, disclosure of their HIV status and/or gender identity, and possible arrest [5, 59]. As a result, transgender women and other key populations may become increasingly reluctant to participate in research or engage with HIV services where healthcare stigma is present, further exacerbating the HIV burden in the region [59].

The findings from this scoping review point to several priorities for future research. Future studies should conduct longitudinal research to better evaluate the effectiveness of interventions in reducing HIV rates, improving mental health and mitigating structural barriers hindering healthcare access. Such research should examine in detail the interplay of stigma, immigration status and religion in transgender women's access to HIV prevention and treatment services offered in the SWANA region, as well as the impact of wars in the region. There is also a need for designing more trans‐specific interventions for transgender women in SWANA, similar to Kaplan et al.’s “Baynetna.” Such interventions should focus on addressing stigma and improving gender affirmation and mental wellbeing among transgender women [14, 44, 45]. As these interventions are developed, their feasibility and acceptability should be evaluated across various populations of transgender women in SWANA [31].

There are some limitations to our scoping review that should be considered. First, our research was restricted to articles written in English or Arabic and pulled from three online databases. We may have missed articles or abstracts published in other languages, such as Persian or Turkish, or published in other databases. Additionally, our relatively narrow search strategy may have introduced selection bias, potentially overrepresenting HIV‐focused studies while omitting broader sexual health, mental health or gender‐affirming care literature that may have been relevant to our aims. Furthermore, although some studies in this review assessed HIV testing measures, none evaluated uptake of or access to PrEP or ART among transgender women in SWANA. It remains unclear whether this gap in our review reflects a limitation of our search strategy or a lack of availability and access to these medications in the region. Prior research indicates that awareness of PrEP is particularly low among transgender women communities compared to other key populations, making this another crucial area for future intervention [46, 47].

## 5 Conclusions

SWANA is one of the few regions in the world where HIV incidence continues to grow at a significant rate [3, 4, 58]. Despite limited research characterizing the HIV burden among transgender women in SWANA, existing evidence suggests they are at an increased risk for HIV acquisition compared to the general population [3, 4]. This scoping review provides an overview of key areas to prioritize in developing future interventions for transgender women in the region. Transgender women in SWANA face numerous barriers to accessing health services, many of which are structural in nature and rooted in a fear of anticipated stigma and violence. Improving healthcare engagement among this population will require tailored programmes and interventions that integrate established frameworks.

In addition, this review highlights several gaps in the literature; most notably, the lack of research centring transgender women as the primary population of interest, stressing the urgent need for longitudinal studies and interventions that are population‐specific. Expanding this research is essential to establishing a more complete understanding of the health needs and healthcare barriers faced by transgender women in SWANA.

## Author Contributions

AK: Software (equal); methodology (equal); formal analysis (lead); Writing – original draft (lead); Writing – review and editing (equal). SAF: Conceptualization (equal); software (equal); methodology (equal); formal analysis (supporting); review and editing (equal). PS: Conceptualization (equal); supervision (lead); review and editing (equal).

## Funding

PS received financial support from the Center for AIDS Prevention Studies, University of California, San Francisco, Mentored Scientist Award (P30AI027763) and the National Institute on Drug Abuse (K24DA061664).

## Conflicts of Interest

The authors declare no conflicts of interest.

## Data Availability

Data sharing is not applicable to this article because it is a review, and no new datasets were generated during the current study.
